# Lifestyle and psychosocial factors and a decline in competence in daily living among Japanese early elderly people: from an age-specified community-based cohort study (NISSIN project)

**DOI:** 10.1186/s12199-019-0787-7

**Published:** 2019-05-06

**Authors:** Satoe Okabayashi, Takashi Kawamura, Kenji Wakai, Masahiko Ando, Kazuyo Tsushita, Hideki Ohira, Shigekazu Ukawa, Akiko Tamakoshi

**Affiliations:** 1 Kyoto University Health Service, Yoshida-Honmachi, Sakyo-ku, Kyoto, 606-8501 Japan; 2 Department of Preventive Medicine, Nagoya University Graduate School of Medicine, 65 Tsurumai-cho, Showa-ku, Nagoya, 466-8550 Japan; 3 Center for Advanced Medicine and Clinical Research, Nagoya University Hospital, 65 Tsurumai-cho, Showa-ku, Nagoya, 466-8550 Japan; 4 Aichi Comprehensive Health Science Center, 1-1 Aza Gengoyama, Oaza Morioka, Higashiura-cho, Chita-gun, Aichi 470-2101 Japan; 5 Department of Psychology, Graduate School of Informatics, Nagoya University, Furo-cho, Chikusa-ku, Nagoya, 464-8601 Japan; 6 Research Unit of Advanced Interdisciplinary Care Science, Graduate School of Human Life Science, Osaka City University, 3-3-138, Sugimoto, Sumiyoshi-ku, Osaka, 558-8585 Japan; 7 Department of Public Health, Faculty of Medicine and Graduate School of Medicine, Hokkaido University, North 15, West 7, Kita-ku, Sapporo, 060-8638 Japan

**Keywords:** Aging, Cohort studies, Lifestyle, Physical functional performance, Risk factors

## Abstract

**Background:**

To let the early elderly live well, understanding how lifestyle and psychosocial factors related to a decline in competence in daily living is important.

**Methods:**

We investigated the associations between lifestyle and psychosocial factors at age 64 years and a decline in the Tokyo Metropolitan Institute of Gerontology Index of Competence score of ≥ 2 points at age 70 years among the participants in comprehensive medical check-ups living in a city in Japan. Multivariable logistic regression analyses were performed separately for men and women.

**Results:**

Of the 1113 eligible men and 1203 eligible women, 110 men and 80 women showed a deteriorated competence in daily living during the 6 years. In men, risk was increased with ≥ 2 nighttime awakenings (multivariable odds ratio [mOR] 2.14, 95% confidence interval [CI] 1.19–3.86) and living alone (mOR 4.68, 95% CI 1.22–18.0), whereas risk was significantly decreased with a medium or fast gait (mOR 0.37 and 0.21, 95% CI 0.21–0.67 and 0.08–0.58) and high academic achievement (mOR 0.32 and 0.43, 95% CI 0.19–0.53 and 0.25–0.72). In women, risk was decreased with high life satisfaction (mOR 0.39, 95% CI 0.16–0.91) and participation in community activities (mOR 0.50, 95% CI 0.29–0.86) but increased with depressive mood (mOR 1.86, 95% CI 1.09–3.18).

**Conclusion:**

Living alone for men and low life satisfaction for women at age 64 years were markedly associated with the risk of a subsequent declining competence in daily living.

## Background

Many developed countries in the world are faced with aging populations. The estimated proportion of people aged ≥ 60 years will increase from 12.3% in 2015 to 21.5% in 2050 in the world [1]. Especially in Japan, the proportion of people aged ≥ 65 years is rapidly increasing year by year and will reach 30% of the population by 2025 [2]. The country is thus considered to be a forerunner of an ever-aging world. Because such an aging population is unprecedented, accommodation to the coming situation is important and urgently required.

Turning 65 is considered to be one of the biggest milestones in life. When people reach this age in Japan, they usually retire from their job if employed, start to draw a state pension, and earn the right to receive nursing care from the social insurance system, regardless of their working status [3]. Such a social system is similar to that of European countries.

From a biological standpoint, most elderly individuals around 65 years of age who live in the community are independent in their activities of daily living (ADL), including eating, dressing, bathing, toileting, transferring, and continence [4, 5]. However, some individuals start to show a declined incompetence in daily living, known as instrumental activities of daily living (IADL), which are necessary to live in the community [5]. IADL directly affects subsequent independent life and well-being in community-dwelling elderly individuals [6], and its decline predicts mortality [7]. It is thus necessary to know the risk or preventive factors related to a decline in competence in daily living at around 65 years of age to help people to live well in later life.

Recent studies have reported several factors causing such a decline in IADL [8–16]. However, the participants in most previous studies were aged ≥ 65 years, with considerable variability in the age range and with few focusing on the early elderly. Because factors associated with a decline in competence in daily living are expected to differ by age, as with mortality [7, 17, 18], we established a cohort of community-dwelling elders with a specified baseline age of 64 years, and we investigated the lifestyle and psychosocial risk and preventive factors in the early elderly related to a decline in competence in daily living.

## Methods

### Study design

The study population was extracted from the New Integrated Suburban Seniority Investigation (NISSIN) project, a community-based prospective cohort study of Japanese elderly individuals of a specified age. Project rationale and design are described elsewhere [19, 20]. Briefly, residents of Nisshin City, Aichi Prefecture, who were 64 years old on January 1 of the respective years from 1996 to 2005 were invited to undergo free-of-charge comprehensive medical check-ups consisted of somatometry, blood tests, and a detailed self-administered questionnaire [20]; 3073 participants (1548 men and 1525 women, 43.9% of eligible residents) who provided informed consent were enrolled in the cohort. They were invited to undergo comprehensive medical check-ups again at age 70 years from 2002 to 2011. Outcomes were measured by the second medical check-ups, home visits by municipal public health nurses, the insurance system for long-term care, or vital statistics.

### Study participants

The subjects of this study were those persons who participated in the medical check-ups at age 64 and whose competence in daily living was not in decline at this baseline.

### Indicator of competence in daily living

As an indicator of competence in daily living, we used the Tokyo Metropolitan Institute of Gerontology Index of Competence (TMIG-IC), a self-administered questionnaire representing the higher dimensional competence in daily living [9, 21]. The TMIG-IC is a multidimensional 13-item index that includes three sublevels of competence: instrumental self-maintenance, intellectual activity, and social role. The response to each item is “yes (score 1)” or “no (score 0)”. The higher the total score, the higher the competence in daily living. The baseline TMIG-IC was measured by the medical check-ups at age 64 years, and follow-up TMIG-IC was measured by the medical check-ups or home visits of public health nurses at age 70 years.

### Lifestyle and psychosocial factors

From the various factors screened at age 64 years, we extracted the lifestyle and psychosocial factors from the self-administered questionnaire that were less missed (< 10%) and were deemed to be biologically associated with the decline in the competence of daily living. They included depressive tendency (shorter version of the Geriatric Depression Scale [GDS]) [22, 23], mental stress or strain, life satisfaction status (life satisfaction index-K) [20], frequency of nighttime awakening, smoking status (never, past, current), frequency of alcohol intake per week, amount of ethanol intake per drink, daily walking time, gait speed, and sleeping hours. Also obtained was information on self-reported working status (current worker or not), participation in community activities, academic background, marital status, and number of family members living together.

### Statistical analysis

We treated a total TMIG-IC score of ≥ 11 points at age 64 years as a preserved competence in daily living. The main outcome was a decline in the competence in daily living of ≥ 2 points in the TMIG-IC score from 64 to 70 years of age. For the analyses, a score of the shorter version of the GDS ≥ 6 was treated as depressive tendency [22, 23]. Life satisfaction status in the life satisfaction index-K questionnaire was divided into three categories (≥ 7, 5 or 6, ≤ 4), and the self-reported frequency of nighttime awakening was also trisected (seldom, about once, about ≥ 2). The following self-reported daily habits were divided into three categories: amount of daily ethanol intake calculated based on the type of beverage, amount per drink, and frequency per week (seldom, < 23 g, ≥ 23 g), daily walking time (< 30 min, 30 min–1 h, ≥ 1 h), and daily sleeping hours (< 6 h, 6–8 h, ≥ 8 h). The activities and participation status in the self-administered questionnaire were treated as follows: working status was dichotomized (current worker or not), and community activity participation was also divided into two categories (participation in ≥ 1 community activity or not) [24]. The academic background was trisected (junior high school, senior high school, junior college, or higher education), marital status was dichotomized (currently married or not), and the number of family members living together was divided into three categories (none, 1, ≥ 2).

Missing variables were replaced with the mean value for continuous data and with the most frequently chosen category for the categorical data in each sex. Baseline characteristics were compared between the sexes using Pearson’s chi-square test or Fisher’s exact test.

To reveal the associations between the baseline lifestyle and psychosocial factors and a subsequent decline in competence in daily living, univariable and multivariable logistic regression analyses were performed in men and women separately and odds ratios (ORs) and their 95% confidence intervals (CIs) were calculated. In the multivariable analysis, we incorporated lifestyle and psychosocial variables any category of which was statistically significant or marginally significant (*P* < .1) in univariable analyses considering the possibility of multicollinearity. Here, we adjusted for the participation year and fundamental medical factors (the present illness of hypertension and past history of cerebrovascular disease in men, no factors in women) that were significantly associated with the outcome in the screening analyses. A trend test was performed when a variable had three ordinal categories. As a sensitivity analysis, we performed analyses in the same way but limited to participants whose baseline TMIG-IC score was 13, the perfect score. All tests of significance were two-tailed, and *P* values of < .05 were considered significant. STATA 12.1 software (STATA Corporation, College Station, TX) was used for statistical analyses.

### Ethical considerations

For informed consent, an opt-out approach was adopted from 1996 to 2001 and individual written informed consent was obtained thereafter [19, 20]. The study was approved by the Ethics Committees of Nagoya University Graduate School of Medicine, the National Center for Geriatrics and Gerontology of Japan, Aichi Medical University of Medicine, and Hokkaido University Graduate School of Medicine.

## Results

**Fig. 1:**
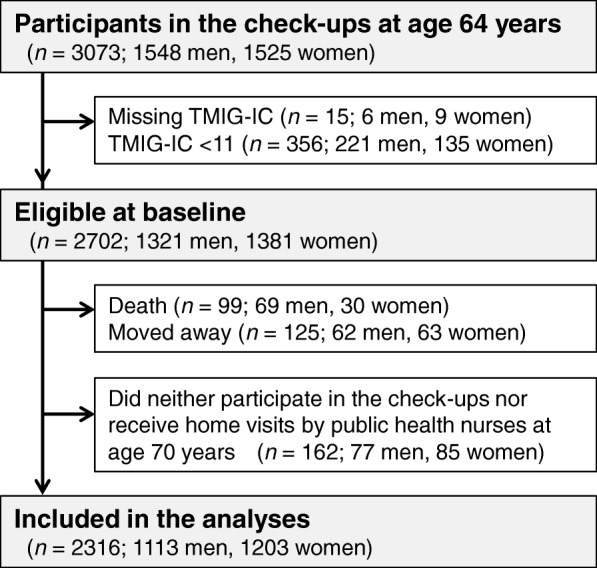
The flow of the study subjects

The study flowchart is shown in Fig. 1. A total of 3073 residents (1548 men and 1525 women) participated in the medical check-ups and consented to data use at age 64 years. We excluded 15 participants (6 men and 9 women) who did not complete the TMIG-IC questionnaire at age 64 years and 356 participants (221 men and 135 women) whose TMIG-IC score was < 11 at age 64 years from this study. Among the 2702 eligible subjects (1321 men and 1381 women), 99 (69 men and 30 women) died and 125 (62 men and 63 women) moved out of the area before reaching age 70 years. After further excluding 162 (77 men and 85 women) who did neither undergo the check-ups nor receive home visits by public health nurses at age 70 years, this left 2316 (1113 men and 1203 women) for analyses.

Baseline lifestyle and psychosocial characteristics of the participants according to sex are shown in Table 1. The proportion of missing data was < 0.3%, except for the frequency of alcohol intake (3.3%). Women were more likely to be depressed and feel stressed than men, but no significant sex difference was found in life satisfaction. The proportion of current smoking, amount of daily ethanol intake, proportions of current working, high final educational attainments, and married status were greater in men, whereas that of living alone was smaller. Walking time was longer but sleeping time was shorter in women.

**Table 1:** Lifestyle and psychosocial characteristics of participants at age 64 years

	Men (*n* = 1113)	Women (*n* = 1203)	*P* value
Mental condition			
Depressive mood^†^	172 (15.5)	259 (21.6)	0.001
Mental strain			
Seldom	610 (55.1)	423 (35.2)	< 0.001
Sometimes	455 (41.1)	686 (57.1)	
Always	42 (3.8)	93 (7.7)	
Life satisfaction^‡^			
Low (≤ 4)	392 (35.3)	441 (36.7)	0.39
Medium (5–6)	422 (38.0)	424 (35.3)	
High (≥ 7)	296 (26.7)	337 (28.0)	
Nighttime awakening			
Seldom	250 (22.5)	249 (20.7)	0.146
Once	463 (41.6)	549 (45.6)	
Twice or more	400 (35.9)	405 (33.7)	
Daily habits			
Smoking			
Never	224 (20.1)	1106 (91.9)	< 0.001
Past	547 (49.2)	62 (5.2)	
Current	341 (30.7)	35 (2.9)	
Daily ethanol intake^§^			
Seldom	341 (31.7)	954 (82.0)	< 0.001
≤ 23 g	460 (42.8)	198 (17.0)	
> 23 g	274 (25.5)	12 (1.0)	
Regular exercise			
Seldom	400 (36.0)	483 (40.2)	0.081
< 1/week	108 (9.7)	98 (8.2)	
≥ 1/week	604 (54.3)	621 (51.7)	
Daily walking time			
< 30 min	182 (16.4)	96 (8.1)	< 0.001
30 min to 1 h	389 (35.1)	305 (25.6)	
≥ 1 h	539 (48.6)	792 (66.4)	
Gait speed			
Slow	97 (8.7)	155 (12.9)	0.003
Medium	872 (78.4)	882 (73.4)	
Fast	143 (12.9)	164 (13.7)	
Daily sleeping hours			
< 6 h	69 (6.2)	154 (12.8)	< 0.001
6–8 h	714 (64.2)	836 (64.5)	
8 h	330 (28.7)	213 (17.7)	
Social activities			
Current working	639 (58.1)	309 (25.9)	< 0.001
Participation in community activities	904 (81.2)	1021 (84.9)	0.019
Environment			
Final educational status			
Junior high school	282 (25.4)	384 (32.0)	< 0.001
Senior high school	452 (40.7)	617 (51.4)	
Junior college or higher	376 (33.9)	199 (16.6)	
Marital status			
Never, widowed, or divorced	42 (3.8)	196 (16.4)	< 0.001
Presently married	1071 (96.2)	1003 (83.7)	
Number of family members living together			
0	13 (1.2)	74 (6.4)	< 0.001
1	540 (48.3)	611 (52.5)	
≥ 2	543 (49.5)	478 (41.1)	

Values are expressed as number (percentage). Percentage was calculated by excluding missing data

^†^Participants whose score on the Geriatric Depression Scale was 6 or more

^‡^Life satisfaction index-K

^§^Mean daily ethanol intake was calculated from the type, amount, and frequency of alcoholic beverages

Table 2 shows the associations of lifestyle and psychosocial factors at age 64 years with a subsequent decline in competence in daily living by univariable and multivariable analyses in men. Of the eligible male participants, 110 (9.9%) showed a deteriorated competence in daily living from age 64 to 70 years. In the univariable analyses, depressive mood, life satisfaction, nighttime awakening, daily ethanol intake, regular exercise, gait speed, final educational status, marital status, and number of family members living together were significantly associated with a decline in competence in daily living. Because of the multicollinearity between marital status and the number of family members living together, we adopted the latter in the multivariable analysis. In the multivariable analysis, risk was significantly increased with ≥ 2 nighttime awakenings (OR 2.14, 95% CI 1.19–3.86) and living alone (OR 4.68, 95% CI 1.22–18.0). Risk was significantly decreased with a medium or fast gait (compared with slow gait, OR 0.37, 95% CI 0.21–0.67 and OR 0.21, 95% CI 0.08–0.58, respectively; *P*_trend_ < .001) and high final educational status (senior high school, junior college, or higher education; compared with junior high school, OR 0.32, 95% CI 0.19–0.53 and OR 0.43, 95% CI 0.25–0.72, respectively; *P*_trend_ = .001). A strong association was found between living alone and a decline in competence in daily living. Of the 13 men living alone, 9 were widowed, 2 were divorced, 1 was currently married, and 1 had never married. Indeed, 4 out of the 13 single men (3 widowed and 1 had never married) showed a decreased competence in daily living at age 70 years.

**Table 2:** Association of lifestyle/psychosocial factors at age 64 years with reduced competence in daily living at age 70 years (males)

	Decline in competence in daily living^†^		Univariable analysis		Multivariable analysis	
	+ (*n* = 110)	− (*n* = 1003)	OR (95% CI)	*P* for trend	OR (95% CI)	*P* for trend
Mental condition						
Depressive mood^‡^	26 (23.6)	146 (14.6)	1.82 (1.13–2.92)		1.14 (0.83–1.96)	
Mental strain						
Seldom	64 (58.2)	552 (55.0)	1	0.962		
Sometimes	39 (35.5)	416 (41.5)	0.81 (0.53–1.23)			
Always	7 (6.4)	35 (3.5)	1.73 (0.74–4.04)			
Life satisfaction^§^						
Low (≤ 4)	54 (49.1)	338 (33.7)	1	0.003	1	0.239
Medium (5–6)	35 (31.8)	390 (38.9)	0.56 (0.36–0.88)		0.73 (0.45–1.20)	
High (≥ 7)	21 (19.1)	275 (27.4)	0.48 (0.28–0.81)		0.73 (0.40–1.32)	
Nighttime awakening						
Seldom	18 (16.4)	232 (23.1)	1	0.003	1	0.005
Once	37 (33.6)	426 (42.5)	1.12 (0.62–2.01)		1.22 (0.66–2.24)	
≥ 2 times	55 (50.0)	345 (34.4)	2.05 (1.18–3.59)		2.14 (1.19–3.86)	
Daily habit						
Smoking						
Never	24 (21.8)	200 (19.9)	1	0.179		
Past	41 (37.3)	507 (50.5)	0.67 (0.40–1.14)			
Current	45 (40.9)	296 (29.5)	1.27 (0.75–2.15)			
Daily ethanol intake^¶^						
Seldom	42 (38.2)	299 (29.8)	1	0.231	1	0.328
≤ 23 g	42 (38.2)	462 (46.1)	0.64 (0.41–1.02)		0.75 (0.46–1.22)	
> 23 g	26 (23.6)	242 (24.1)	0.76 (0.46–1.28)		0.78 (0.45–1.35)	
Regular exercise						
Seldom	53 (48.2)	347 (34.6)	1	0.007	1	0.122
< 1/week	9 (8.2)	99 (9.9)	0.60 (0.28–1.25)		0.85 (0.39–1.83)	
≥ 1/week	48 (43.6)	557 (55.5)	0.56 (0.37–0.85)		0.70 (0.45–1.10)	
Daily walking time						
< 30 min	19 (17.3)	163 (16.3)	1	0.756		
30 min to 1 h	39 (35.5)	353 (35.2)	0.95 (0.53–1.69)			
≥ 1 h	52 (47.3)	487 (48.6)	0.92 (0.53–1.59)			
Gait speed						
Slowly	21 (19.1)	76 (7.6)	1	< 0.001	1	< 0.001
Medium	83 (75.5)	790 (78.8)	0.38 (0.22–0.65)		0.37 (0.21–0.67)	
Faster	6 (5.5)	137 (13.7)	0.16 (0.06–0.41)		0.21 (0.08–0.58)	
Daily sleeping hours						
< 6 h	11 (10.0)	58 (5.8)	1.77 (0.89–3.54)	0.216		
6–8 h	69 (62.7)	645 (64.3)	1			
≥ 8 h	30 (27.3)	300 (29.9)	0.93 (0.60–1.47)			
Social activities						
Current worker	64 (58.2)	589 (58.7)	0.98 (0.66–1.46)			
Participation in community activities	88 (80.0)	816 (81.4)	0.92 (0.56–1.50)			
Environment						
Final educational status						
Junior high school	52 (47.3)	230 (22.9)	1	< 0.001	1	0.001
Senior high school	29 (26.4)	425 (42.4)	0.30 (0.19–0.49)		0.32 (0.19–0.53)	
Junior college or higher	29 (26.4)	348 (34.7)	0.37 (0.23–0.60)		0.43 (0.25–0.72)	
Marital status						
Never, widowed, or divorced	8 (7.3)	34 (3.4)	1			
Presently married	102 (92.7)	969 (96.6)	0.45 (0.20–0.99)			
Number of family members living together						
0	4 (3.6)	9 (0.9)	4.61 (1.37–15.5)	0.905	4.68 (1.22–18.0)	0.665
1	49 (44.5)	508 (50.6)	1		1	
≥ 2	57 (51.8)	486 (48.5)	1.22 (0.81–1.82)		1.09 (0.71–1.67)	

OR, 95% CI, and *P* for trend were calculated by using imputation to provide the missing data. Multivariable analysis: adjusted for year of participation and history of hypertension or cerebrovascular disease

*OR* odds ratio, *CI* confidence interval

^†^Reduced score on the Tokyo Metropolitan Institute of Gerontology Index of Competence of ≥ 2 from 64 years of age

^‡^Geriatric Depression Scale ≥ 6

^§^Life satisfaction index-K

^¶^Mean daily ethanol intake was calculated from the type, amount, and frequency of alcoholic beverages

Table 3 shows the associations of lifestyle and psychosocial factors at age 64 years with a subsequent decline in competence in daily living by univariable and multivariable analyses in women. Of the eligible female participants, 80 (6.7%) showed a deteriorated competence in daily living over the 6 years. In the univariable analyses, a decline was significantly associated with depressive mood, mental stress status, life satisfaction, nighttime awakening, regular exercise, gait speed, daily sleeping hours, participation in community activities, and final educational status. There was no significant multicollinearity among these factors. In the multivariable analysis, the risk of reduced competence in daily living was significantly increased with depressive mood (OR 1.86, 95% CI 1.09–3.18) and significantly decreased with high life satisfaction (OR 0.39, 95% CI 0.16–0.91) and participation in community activities (OR 0.50, 95% CI 0.29–0.86). A decreased risk was marginally significantly associated with regular exercise (≥ 1 time per week) (OR 0.63, 95% CI 0.38–1.07).

**Table 3:** Association of lifestyle/psychosocial factors at age 64 years with reduced competence in daily living at age 70 years (females)

	Decline in competence in daily living^†^		Univariable analysis		Multivariable analysis	
	+ (*n* = 80)	− (*n* = 1123)	OR (95% CI)	*P* for trend	OR (95% CI)	*P* for trend
Mental condition						
Depressive mood^‡^	35 (43.8)	224 (18.3)	3.12 (1.96–4.97)		1.86 (1.09–3.18)	
Mental strain						
Seldom	24 (30.0)	399 (32.6)	1	0.033	1	0.628
Sometimes	43 (53.8)	644 (52.7)	1.11 (0.66–1.86)		0.85 (0.49–1.49)	
Always	13 (16.3)	80 (6.5)	2.70 (1.32–5.53)		1.43 (0.63–3.24)	
Life satisfaction^§^						
Low (≤ 4)	44 (55.0)	397 (32.5)	1	< 0.001	1	0.054
Medium (5–6)	28 (35.0)	397 (32.5)	0.64 (0.39–1.04)		0.98 (0.56–1.72)	
High (≥ 7)	8 (10.0)	329 (26.9)	0.22 (0.10–0.47)		0.39 (0.16–0.91)	
Nighttime awakening						
Seldom	12 (15.0)	237 (19.4)	1	0.044	1	0.314
Once	33 (41.3)	516 (42.2)	1.26 (0.64–2.49)		1.35 (0.67–2.71)	
≥ 2 times	35 (43.8)	370 (30.3)	1.87 (0.95–3.67)		1.46 (0.72–2.96)	
Daily habit						
Smoking						
Never	75 (93.8)	1031 (84.3)	1	0.602		
Past	3 (3.8)	59 (4.8)	0.70 (0.21–2.28)			
Current	2 (2.5)	33 (2.7)	0.83 (0.20–3.54)			
Daily ethanol intake^¶^						
Seldom	69 (86.3)	885 (72.4)	1	0.092		
< 23 g	11 (13.8)	226 (18.5)	0.62 (0.32–1.20)			
≥ 23 g	0 (0.0)	12 (1.0)	1			
Regular exercise						
Seldom	44 (55.0)	439 (35.9)	1	0.001	1	0.093
< 1/week	9 (11.3)	89 (7.3)	1.01 (0.48–2.14)		1.30 (0.59–2.86)	
≥ 1/week	27 (33.8)	595 (48.7)	0.45 (0.28–0.74)		0.63 (0.38–1.07)	
Daily walking time						
< 30 min	8 (10.0)	88 (7.2)	1	0.147		
30 min to 1 h	25 (31.3)	280 (22.9)	0.98 (0.43–2.26)			
≥ 1 h	47 (58.8)	755 (61.7)	0.68 (0.31–1.50)			
Gait speed						
Slowly	16 (20.0)	139 (11.4)	1	0.005	1	0.196
Medium	60 (75.0)	824 (67.4)	0.63 (0.35–1.13)		0.92 (0.50–1.71)	
Faster	4 (5.0)	160 (13.1)	0.22 (0.07–0.66)		0.41 (0.13–1.30)	
Daily sleeping hours						
< 6 h	16 (20.0)	138 (11.3)	1.90 (1.05–3.45)	0.409	1.58 (0.84–2.98)	0.753
6–8 h	48 (60.0)	788 (64.4)	1		1	
≥ 8 h	16 (20.0)	197 (16.1)	1.33 (0.74–2.40)		1.37 (0.74–2.55)	
Social activities						
Current worker	19 (23.8)	290 (23.7)	0.89 (0.53–1.52)			
Participation in community activities	55 (68.8)	966 (79.0)	0.36 (0.22–0.59)		0.50 (0.29–0.86)	
Environment						
Final educational status						
Junior high school	38 (47.5)	346 (28.3)	1	0.029	1	0.63
Senior high school	29 (36.3)	588 (48.1)	0.45 (0.27–0.74)		0.64 (0.38–1.09)	
Junior college or higher	13 (16.3)	189 (15.5)	0.63 (0.33–1.20)		1.04 (0.52–2.10)	
Marital status						
Never, widowed, or divorced	14 (17.5)	182 (14.9)	1			
Presently married	66 (82.5)	941 (76.9)	0.91 (0.50–1.66)			
Number of family members living together						
0	5 (6.3)	69 (5.6)	1.02 (0.39–2.67)	0.979		
1	43 (53.8)	608 (49.7)	1			
≥ 2	32 (40.0)	466 (38.1)	0.89 (0.63–1.63)			

OR, 95% CI, and *P* for trend were calculated by using imputation to provide the missing data. Multivariable analysis: adjusted for year of participation

*OR* odds ratio, *CI* confidence interval

^†^Reduced score on the Tokyo Metropolitan Institute of Gerontology Index of Competence of ≥ 2 from 64 years of age

^‡^Geriatric Depression Scale ≥ 6

^§^Life satisfaction index-K

^¶^Mean daily ethanol intake was calculated from the type, amount, and frequency of alcoholic beverages

When limited to participants whose TMIG-IC was 13 at age 64 years, the associations of psychosocial factors at age 64 years with a subsequent decline in competence in daily living were essentially unaltered for both men and women.

## Discussion

To our knowledge, this is the first study to show the risk and preventive factors in early elderly individuals related to a subsequent decline in competence in daily living. The strong point of this study is all the participants were the same age, 64 years old. By focusing the subjects' age at 64 years, we could overcome the limitation of the previous studies that the age was widely distributed which strongly affected the incidence of outcomes.

For men, living alone was associated with a low competence in daily living in previous cross-sectional studies only, which do not allow inference of causal relationships [25, 26]. Our cohort study showed a very strong association between living alone and a subsequent decline in competence in daily living, suggesting a cause-and-effect relationship. There may be two major reasons for this. First, men tend not to be familiar with cooking and are more likely to be indifferent with regard to nutritional practice [27–29]. Men who live alone, therefore, might not eat healthily, which would impact their health and affect their competence in daily living [29]. Second, men are less integrated within their social networks [30, 31]. A qualitative study speculated that Japanese elderly men tended to refuse to socialize widely because of embarrassment socializing with women but would be comfortable talking with small numbers of men in the same age bracket [32]. Men who live alone, therefore, might get less social support, leading to poor outcomes. Unfortunately, we could not analyze the decline in competence according to the reason such as marital status for living alone because of the small number of men in the respective reason categories.

Life satisfaction is well known to be related to functional ability [33, 34]. Previous cohort studies also showed associations between life satisfaction and mortality in elderly people [35, 36]. However, it is unknown whether the life satisfaction level modifies functional ability. Although their causal relationship is unclear, higher life satisfaction might induce relatively active health behaviors and prevent the deterioration in health status [36]. However, such an association was found only in women [35, 37]. According to the studies by Bowling and Grundy reporting life satisfaction and mortality, women’s affinity with social networks would improve life satisfaction and play a protective role [35, 37]. Among Japanese elderly individuals, a larger number of friends and social activities also enhance life satisfaction in women but not in men [38]. Hence, women are more sensitive to social relations than men.

The other findings that participation in community activities [8, 10–12], physical activity [8, 14], a small-to-moderate amount of ethanol intake [8, 15], and high academic achievement [16] were negatively associated with a decline in competence in daily living but that depressive mood [8, 39] and poor quality of sleep [13] were positively associated were consistent with those of previous cohort studies. However, those findings were obtained from older people with a wide age range. Here, we have provided new evidence obtained specifically from early elderly individuals that could help people to improve their well-being in later life. Moreover, most past studies evaluated simple IADL as the outcome. We used TMIG-IC, a more comprehensive scale including instrumental self-maintenance, intellectual activity, and social role, which allowed us to capture the delicate decline in competence in daily living.

There are three limitations to this study. Firstly, except the participants who died before age 70 years, 11.0% of the participants at baseline could not be followed at 70 years because they moved out (4.8%) or failed to receive neither the on-site health check-ups nor the public health nurses’ home visits (6.2%). Individuals with a deteriorated competence in daily living could not take part in the follow-up health check-up at age 70 years, which might cause some selection biases. However, the baseline characteristics of the unfollowed participants were not much different from those of the participants of the present analyses. Secondly, we could not include important potential confounders such as cognition, hearing, and vision, because these data were not systematically collected. However, we consider the participants were apparently healthy without terrible dysfunctions in cognition, hearing, and vision, because the participants of this research were all community-dwelling and walking in the health check-up at age 64 years. Lastly, it was impossible to obtain the outcome at age 70 years for the participants who died before 70 years of age. However, the directions of the risk/preventive factors for the decline in competence of daily living were not different by vital status in our preliminary analyses.

Because middle-aged and presenile adults are interested in maintaining their health, our results would be useful to them. In addition, our findings offer some ideas to healthcare providers and policymakers when they consider what to do for early elderly individuals or a little younger ones in order to help them to live well in their later years. By referring to our study results, healthcare workers could provide important information to the public and supply necessary care or services to those individuals who will benefit most.

## Conclusion

In addition to known factors, living alone for men and low life satisfaction for women at age 64 years were markedly associated with the risk of declining competence in daily living during the subsequent 6 years among community-dwelling Japanese people.

## Abbreviations

ADL: Activities of daily living
CI: Confidence interval
GDS: Geriatric Depression Scale
IADL: Instrumental activities of daily living
OR: Odds ratio
TMIG-IC: Tokyo Metropolitan Institute of Gerontology Index of Competence
